# On the importance of parenting in externalizing disorders: an evaluation of indirect genetic effects in families

**DOI:** 10.1111/jcpp.13654

**Published:** 2022-07-02

**Authors:** Espen M. Eilertsen, Rosa Cheesman, Ziada Ayorech, Espen Røysamb, Jean‐Baptiste Pingault, Pål R. Njølstad, Ole A. Andreassen, Alexandra Havdahl, Tom A. McAdams, Fartein A. Torvik, Eivind Ystrøm

**Affiliations:** ^1^ Department of Psychology, PROMENTA Research Center University of Oslo Oslo Norway; ^2^ Centre for Fertility and Health Norwegian Institute of Public Health Oslo Norway; ^3^ Division of Psychology and Language Sciences University College London London UK; ^4^ MRC Social, Genetic and Developmental Psychiatry Centre Institute of Psychiatry, King's College London UK; ^5^ Department of Clinical Science, Center for Diabetes Research University of Bergen Bergen Norway; ^6^ Children and Youth Clinic Haukeland University Hospital Bergen Norway; ^7^ Division of Mental Health and Addiction, NORMENT Oslo University Hospital Oslo Norway; ^8^ Institute of Clinical Medicine University of Oslo Oslo Norway; ^9^ Department of Mental Disorders Norwegian Institute of Public Health Oslo Norway; ^10^ Nic Waals Institute, Lovisenberg Diaconal Hospital Oslo Norway; ^11^ Social, Genetic and Developmental Psychiatry Centre Institute of Psychiatry, Psychology and Neuroscience, King's College London London UK; ^12^ School of Pharmacy University of Oslo Oslo Norway

**Keywords:** Externalizing disorders, parenting, indirect genetic effects, gene–environment correlation, MoBa

## Abstract

**Background:**

Theoretical models of the development of childhood externalizing disorders emphasize the role of parents. Empirical studies have not been able to identify specific aspects of parental behaviors explaining a considerable proportion of the observed individual differences in externalizing problems. The problem is complicated by the contribution of genetic factors to externalizing problems, as parents provide both genes and environments to their children. We studied the joint contributions of direct genetic effects of children and the indirect genetic effects of parents through the environment on externalizing problems.

**Methods:**

The study used genome‐wide single nucleotide polymorphism data from 9,675 parent–offspring trios participating in the Norwegian Mother Father and child cohort study. Based on genomic relatedness matrices, we estimated the contribution of direct genetic effects and indirect maternal and paternal genetic effects on ADHD, conduct and disruptive behaviors at 8 years of age.

**Results:**

Models including indirect parental genetic effects were preferred for the ADHD symptoms of inattention and hyperactivity, and conduct problems, but not oppositional defiant behaviors. Direct genetic effects accounted for 11% to 24% of the variance, whereas indirect parental genetic effects accounted for 0% to 16% in ADHD symptoms and conduct problems. The correlation between direct and indirect genetic effects, or gene–environment correlations, decreased the variance with 16% and 13% for conduct and inattention problems, and increased the variance with 6% for hyperactivity problems.

**Conclusions:**

This study provides empirical support to the notion that parents have a significant role in the development of childhood externalizing behaviors. The parental contribution to decrease in variation of inattention and conduct problems by gene–environment correlations would limit the number of children reaching clinical ranges in symptoms. Not accounting for indirect parental genetic effects can lead to both positive and negative bias when identifying genetic variants for childhood externalizing behaviors.

## Introduction

The neurodevelopmental and disruptive disorders ADHD, conduct disorder (CD) and oppositional defiant disorder (ODD) can be grouped under the term childhood externalizing disorders (Liu, 2004). Theoretical models emphasize a complex interplay of genes and environments in the development of childhood externalizing disorders (Beauchaine & McNulty, 2013; Liu, 2004). In their ontogenic process model, Beauchaine and McNulty (2013) view individual differences in externalizing disorders as resulting from polygenic liabilities that interact with environmental risk factors through development.

Studies of twins have supported a prominent role of genetic influences to individual differences in childhood externalizing disorders. Bornovalova, Hicks, Iacono, and McGue (2010) estimated heritability coefficients of 73%, 51% and 73% for ADHD, CD and ODD, respectively. Twin studies have also supported the view that comorbidity among the specific disorders can be ascribed to a common genetic liability (Bornovalova et al., 2010; Tuvblad, Zheng, Raine, & Baker, 2009). Heritability estimates based on common single nucleotide polymorphisms (SNPs) are somewhat inconsistent across studies, ranging between 5% and 46% (Cheesman et al., 2017; Karlsson Linnér et al., 2021; Mollon et al., 2021; Pappa et al., 2015). Although genetic variation appears to play an important contribution to individual differences, little is known about the biology underlying externalizing problems (Barr & Dick, 2019).

With respect to environmental contributions to externalizing disorders, aspects of the family environment have been hypothesized to play an important role through development (Beauchaine & McNulty, 2013). In a meta‐analysis of family studies, Burt (2009) reported that at age 6–10 years, 23% of the variability in externalizing problems could be attributed to environmental effects that are shared among siblings. Such between‐family effects may partially reflect effects of parental behaviors, but also other aspects of the environment such as peers or schools. These estimates are informative of the net importance of shared environments because they do not require that the relevant factors are known and measured. Several empirical studies have investigated the importance of specific familial risk factors. These include maternal depression (Blatt‐Eisengart, Drabick, Monahan, & Steinberg, 2009; Edwards & Hans, 2015; Gjerde et al., 2017), hostile parenting (Edwards & Hans, 2015), parental involvement (Beyers, Bates, Pettit, & Dodge, 2003), parental alcohol use (Lund et al., 2020; Rossow, Felix, Keating, & McCambridge, 2016) and parental education (Torvik et al., 2020).

Although several aspects of the family environment have been implicated in externalizing disorders, individual effects are typically of small effect and cannot explain much of the observed differences among individuals. Beauchaine and McNulty (2013) consider bidirectional transactions with multiple parental behaviors that may change and accumulate through development as important pathways for externalizing behaviors. From this perspective, any specific aspect of the family environment is not expected to account for much of the individual differences in externalizing disorders. This poses a difficult methodological challenge related to measuring and characterizing all relevant parental behaviors.

Another challenge with studying the importance of the family environment is that parents provide both genes and environment for their children. Parental behaviors and childhood externalizing disorders may therefore correlate irrespective of any direct effect of parents through the environment (Knafo & Jaffee, 2013). Multiple sources of evidence indicate pleiotropy (the same genes affect multiple traits) across a broad range of human traits (Bulik‐Sullivan et al., 2015). Pleiotropic effects of genes could lead to various parental behaviors becoming correlated with externalizing problems. For instance, children who inherit a genetic disposition towards externalizing problems may also experience an adverse environment, because the same genes that predispose for externalizing problems may also affect parents' ability to provide a supportive environment. This would induce a positive association between children genetic predisposition to externalizing problems and the impact of the family environment, known as a gene–environment correlation (Eaves, Last, Martin, & Jinks, 1977; Plomin, DeFries, & Loehlin, 1977). Alternatively, children who inherited a genetic predisposition towards externalizing problems may also evoke parental attempts to counteract such behaviors, thereby providing an environment that pushes towards a normative behavior pattern. This could induce a negative correlation between children genetic predisposition to externalizing problems and the impact of the family environment. Such gene–environment correlations vitiate any attempt to quantify the importance family environments without a joint consideration of genetic effects. Two review studies concluded that gene–environment correlations were prevalent across a range of mental health problems in children (Jami, Hammerschlag, Bartels, & Middeldorp, 2021; McAdams et al., 2014).

Plomin and Daniels (1987) postulated that investigations on the compound of stochastic events specific to an individual would ‘likely to prove a dead end for research’ and therefore considered ‘a gloomy prospect’. The assumption is that the universe of idiosyncratic events is indefinite and of great aggregate importance as ‘environments’ in twin and family studies, but a Sisyphean task to capture. To address this difficulty, we used an alternative approach to measure the importance of parents on externalizing problems that avoid the need for measuring the relevant parental characteristics. This approach relies on the assumption that relevant parental characteristics are themselves heritable, like most other human traits, characteristics and behaviors (Polderman et al., 2015; Turkheimer, 2000). For example, in a review of the literature, Kendler and Baker (2007) reported a heritable component across a range of parenting behaviors such as warmth, control and protectiveness. By measuring the whole genome of the parents, we could indirectly index *all* heritable parenting traits, characteristics or behaviors relevant to externalizing problems, regardless of whether these are measurable or even known to us. When individuals' genes affect the trait of others, it is known as an indirect genetic effect (Bijma, 2014). Eilertsen et al. (2021) recently described a method for measuring the extent that children traits depend directly on their own genes and indirectly on their parents' genes based on genome wide single nucleotide polymorphism (SNP) data.

Here, we estimate indirect genetic effects to address the importance of parental contributions to externalizing disorders while accounting for some of the challenges imposed by the interplay of genes and environment through development. We study symptoms of attention‐deficit/hyperactivity disorder, oppositional defiant disorder and conduct disorder at 8 years of age, in genotyped parent–offspring trios participating in the Norwegian Mother, Father and Child Cohort study.

## Methods

### Sample

The study is based on data from the Norwegian Mother, Father and Child Cohort Study (MoBa, Magnus et al., 2016). MoBa is a population‐based pregnancy cohort study conducted by the Norwegian Institute of Public Health. Participants were recruited from all over Norway from 1999 to 2008. The women consented to participation in 41% of the pregnancies. The cohort now includes 114,500 children, 95,200 mothers and 75,200 fathers. This study is based on version 12 of the quality‐assured data files released for research in 2020. The establishment of MoBa and initial data collection were based on a license from the Norwegian Data Protection Agency and approval from The Regional Committees for Medical and Health Research Ethics. The MoBa cohort is now based on regulations related to the Norwegian Health Registry Act. This study was approved by The Regional Committees for Medical and Health Research Ethics (project# 2013/863). Further details on the MoBa study and how to obtain a data access can be found at https://www.fhi.no/en/studies/moba/.

### Genotype quality control

The current MoBa genomic dataset comprises imputed genetic data for 98,110 individuals (~32,000 parent–offspring trios; before quality control), derived from nine batches of participants, who make up four study cohorts. Within each batch, parent and offspring genetic data were quality controlled separately. Pre‐imputation quality control criteria have been described in previous publications and are detailed in the Supporting Information. We conducted post‐imputation quality control, retaining SNPs meeting the following criteria: imputation quality score ≥0.8 in all batches, non‐duplicated (by position or name), call rate >98%, minor allele frequency >1%, Hardy–Weinberg equilibrium *p* < .001, not associated with genotyping batch at the genome‐wide level, and not causing a Mendelian error. We removed individuals with the following criteria: heterozygosity outliers (F‐het ±0.2), call rate <98%, reported sex mismatching SNP‐based sex, duplicates (identified using PLINK's (Chang et al., 2015) –genome command as having pihat ≥0.98, and distinguished from monozygotic twins through linkage to unique IDs in the population register, plus age, sex and kinship information within MoBa), individuals with excessive numbers of close relatives (cryptic relatedness) and Mendelian errors. To minimize environmental confounding, we identified a sub‐sample of individuals with European ancestries via principal component analysis using the 1,000 Genomes reference; thresholds for exclusion of outliers were based on visual inspection of a plot of principal components 1 and 2. We used solely SNP data, not self‐reported categorical information, to identify the subsample of European‐ancestry individuals. Exclusions based on visual inspection of the first two principal components led to the reduction of the sample from 98,110 to 97,496 (614 individuals excluded). The final numbers of individuals and SNPs passing quality control were 93,582 and 6,797,215, respectively.

### Selection of parent–offspring trios

The quality control of genotype data retained 25.332 complete parent‐offspring trios. In families with multiple children, we selected one individual at random. Out of these, 11,560 trios participated in the data collection wave when the response data were obtained. This decrease in sample size is mainly due to attrition as the response data were collected when the children were 8 years of age, whereas the genotype data was collected at birth. We refer to Magnus et al. (2016) for a description of attrition in the MoBa study. Our main analyses (described below) relies on identifying different types of genetic effects based on genetic relatednesss within and between parent–offspring trios. We computed an empirical estimate of the genetic relatedness among all individuals, referred to as a genomic relatedness matrix (GRM; Yang, Lee, Goddard, & Visscher, 2011). Closely related individuals can disproportionally influence genetic variance estimates and introduce confounding from environmental effects not specified in the model (Yang, Zeng, Goddard, Wray, & Visscher, 2017). We used a threshold of 0.10 for the largest allowed genetic correlation between any two individuals (ignoring parent–offspring pairs), reasoning that this will exclude most relations likely to share environments without substantially reducing the sample size. Computation of the GRM and selection of individuals using the ‘bottom up’ algorithm, was done with functions from the OpenMendel project (Zhou et al., 2020). After selection of individuals, 9,675 parent–offspring trios had available response data.

### Measures

Measures of externalizing behaviors were obtained from maternal reports on the Rating Scale for Disruptive Behaviors (RS‐DBD; Silva et al., 2005) when the children were 8 years of age. The rating scale includes 8 items related to CD, 9 items related to inattention aspects of ADHD, 9 items related to hyperactivity aspects of ADHD and 8 items related to ODD. We use conduct, inattention, hyperactivity and oppositional defiant to refer to the individual subscales. The items were rated on a scale from 1 to 4 reflecting the frequency of different behaviors. For the analysis, we summed the individual items within each subscale. For individuals with less than 50% missing data on either scale, missing item values were imputed by the sample mean. Otherwise, they were excluded from analyses. Across subscales, 97.9%–98.6% of the participants had complete responses to all items. The imputation procedure resulted in less than 0.5% of missing individuals for either of the subscales. In Table S1, we show the distribution of missing responses for each subscale.

### Analyses

We applied the method described in Eilertsen et al. (2021) for measuring the extent that children phenotypes depend indirectly on additive effects of their parents' genotypes, in addition to directly on additive effects of their own genotypes. The direct and indirect genetic effects here represent the combined influence of all genetic variants tagged by genotyped and imputed SNPs. The model can be viewed as a multiple regression model for the offspring phenotype

$$y=g_{o}+g_{m}+g_{p}+\epsilon .$$

$g_{o}$ represents the direct genetic effect originating in the offspring, $g_{m}$ and $g_{p}$ represents the indirect maternal and paternal genetic effects and ϵ residuals (containing all other sources of variability). The different genetic effects are assumed to be correlated across individuals according to their GRM values. Our interest lies in quantifying the variance of direct genetic effects ($\sigma_{o}^{2}$), indirect maternal and paternal genetic effects ($\sigma_{m}^{2}$ and $\sigma_{p}^{2}$), the covariance between direct and indirect maternal and paternal genetic effects ($\sigma_{\mathrm{om}}$ and $\sigma_{\mathrm{op}}$), the covariance between indirect maternal and paternal genetic effects ($\sigma_{\mathrm{mp}}$) and the residuals ($\sigma_{\epsilon }^{2}$) . The covariance terms are necessary if partially the same genes contribute directly and indirectly. Because parents and offspring are related, a positive covariance between direct and indirect genetic effects will increase variability in the phenotype whereas a negative covariance will decrease variability. We refer to this specification as ‘Differential parental’ as we differentiate between maternal and paternal effects. This can be useful if the importance of maternal and paternal contributions differs, or if different behaviors mediate the indirect genetic effects. Usually, the covariance between two components contributes with a factor of two to the total variance. However, because parents are not related, the covariance between indirect effects is not expected to contribute to the variance. And, as parents and offspring are one generation apart and the expected genetic relatedness between parents and offspring is 1/2, the variance accounted for by covariance between indirect and direct genetic effects is equal to the covariance. The expected variance of the phenotype is equal to $\sigma_{o}^{2}+\sigma_{m}^{2}+\sigma_{p}^{2}+\sigma_{\mathrm{om}}+\sigma_{\mathrm{op}}+\sigma_{\epsilon }^{2}$. The left panel in diagram 1 illustrates the assumed configuration of effects within a parent–offspring trio.

**Figure 1:**
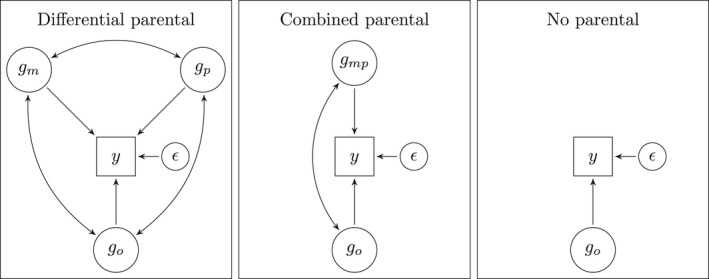
Illustration of the model structure for the alternative specifications of genetic effects. The rectangles represent the observed values on externalizing behaviors, whereas the circles represent the total genetic influence directly from offspring ($g_{o}$), indirectly from mothers ($g_{m}$), indirectly from fathers ($g_{p}$), combined from both parents ($g_{\mathrm{mp}}$) and residuals (ϵ)

We fitted three alternative, simpler models of how children's phenotypes may depend on genetic effects, nested within the ‘Differential parental’ model. It could be sufficient to consider only the combined parental contribution, for example if mostly the same behaviors are important irrespective of the sex of the parents. This is obtained by substituting the maternal and paternal genetic effect with a combined parental genetic effect $g_{\mathrm{mp}}=g_{m}+g_{p}$. This model, previously used by Young et al. (2018) implies that maternal and paternal genetic effects are of equal importance and perfectly correlated $\left(\sigma_{m}^{2}=\sigma_{p}^{2}=\sigma_{\mathrm{mp}}\right)$, and equally correlated with the direct genetic effect $\left(\sigma_{\mathrm{om}}=\sigma_{\mathrm{op}}\right)$. The variance accounted for by the combined genetic effect is then equal to $\sigma_{m}^{2}+\sigma_{p}^{2}$ and the covariance with direct genetic effect equal to $\sigma_{\mathrm{om}}+\sigma_{\mathrm{op}}$. We refer to this as the ‘Combined parental’ model, which is illustrated in the middle panel of Figure 1. We also considered a model without any indirect genetic effects of parents. This is equivalent to the genomic‐relatedness‐matrix restricted maximum likelihood method, implemented in GCTA software (Yang et al., 2011). This is illustrated in the right panel of Figure 1 as ‘No parental’. For comparison, we include the results from a ‘Null’ model without any genetic effects.

We fitted these four alternative specifications to each of the four externalizing subscales separately. In all analyses we additionally included an intercept term and a main effect of child's sex as males and females typically differ in the average levels of externalizing behaviors. The models were estimated using the Julia programming language (Bezanson, Edelman, Karpinski, & Shah, 2017), via the package VCModels.jl (Eilertsen, 2021).

## Results

### Sample characteristics

Table 1 displays characteristics of the study sample. The average age of mothers was 39 years with a standard deviation of 4.3 years. Fathers were on average around 2 years older than mothers, but also more variable with a standard deviation of 5.1 years. Among mothers, 75.9% had completed at least undergraduate tertiary education, whereas 55.2% of the fathers had completed the same level of education. Among the children, males and females where approximately equally represented. Most of the families (92.9%) had more than one child in the household.

**Table 1:** Characteristics of the study sample

Variable	Statistic
Age mothers	
Mean	39.0
Standard deviation	4.3
Minimum	24
Maximum	55
Age fathers	
Mean	41.4
Standard deviation	5.1
Minimum	27
Maximum	76
Education mothers	
% Lower secondary	3.3
% Upper secondary	19.1
% Post‐secondary non‐tertiary	1.7
% First stage of tertiary, undergraduate level	55.3
% First stage of tertiary, graduate	19.1
% Second stage of tertiary, postgraduate	1.5
Education fathers	
% Lower secondary	6.3
% Upper secondary	32.9
% Post‐secondary non‐tertiary	5.7
% First stage of tertiary, undergraduate level	34.4
% First stage of tertiary, graduate	18.5
% Second stage of tertiary, postgraduate	2.3
Gender children	
% Male	51.5
% Female	48.5
Number of children in household	
% 1	7.1
% 2	52.5
% 3	34.5
% 4 or more	5.9

### Genetic analyses

Table 2 summarizes results from the genetic analyses of each subscale. Parameter estimates are standardized so that the total variance after conditioning on sex equals one. Looking at the AIC statistics, it appears that all the models involving genetic effects provide a (substantial) improvement over the ‘Null’ model (AIC values are lower for the genetic models). However, there is no strong evidence supporting either of the genetic models. The ‘Combined parental’ model is always favored over the more elaborate ‘Differential parental’ model, and only for oppositional defiant is the ‘No parental’ model favored. A similar pattern is indicated from a set of likelihood ratio tests sequentially comparing the less elaborate models. This is presented in the *p*‐value column of Table 2. At a 5% level, there is no significant loss of fit by considering the ‘Combined parental’ models, but for conduct and hyperactivity, there is a significant loss of fit by considering the ‘No parental’ models. Because the parameters in Table 2 are standardized, $1-\sigma_{\epsilon }^{2}$ can be seen as a measure of the total variance explained by direct and indirect genetic effects. The corresponding values are larger for more complex models. Considering the models with lowest AIC values, the variance explained was 17%, 27%, 25% and 12% for conduct, inattention, hyperactivity and oppositional defiant, respectively. The variance explained may include components attributable to direct and indirect genetic effects as well as covariances that may be positive or negative, and it is of interest to inspect the individual components of variance.

**Table 2:** Parameter estimates and fit statistics for all subscales for each model specification

Model	Parameter							−2ll	AIC	*df*	*p*‐Value
	$\sigma_{o}^{2}$	$\sigma_{m}^{2}$	$\sigma_{p}^{2}$	$\sigma_{\mathrm{om}}$	$\sigma_{\mathrm{op}}$	$\sigma_{\mathrm{mp}}$	$\sigma_{\epsilon }^{2}$				
Conduct											
Differential	.266	.059	.137	−.114	−.173	.090	.824	27,403.48	27,421.48	7	
Combined	.191	.071	.071	−.083	−.083	.071	.831	27,406.86	**27,418.86**	4	.34
No parental	.077						.923	27,413.43	27,421.43	2	.04
Null							1.00	27,417.56	27,423.56	1	.04
Inattention											
Differential	.238	.061	.144	−.020	−.107	.062	.685	27,605.66	27,623.66	7	
Combined	.240	.082	.082	−.064	−.064	.082	.726	27,608.97	**27,620.97**	4	.35
No parental	.199						.801	27,614.01	27,622.01	2	.08
Null							1.00	27,641.47	27,647.47	1	<.01
Hyperactivity											
Differential	.115	.054	.041	.037	.025	.029	.728	27,536.51	27,554.51	7	
Combined	.116	.039	.039	.030	.030	.039	.746	27,536.99	**27,548.99**	4	.92
No parental	.221						.779	27,544.64	27,552.64	2	.02
Null							1.00	27,579.26	27,585.26	1	<.01
Oppositional defiant											
Differential	.183	.059	.038	−.028	−.069	.035	.818	27,538.14	27,556.14	7	
Combined	.122	.000	.000	.000	.000	.000	.878	27,541.81	27,553.81	4	.30
No parental	.122						.878	27,541.81	**27,549.81**	2	1.00
Null							1.00	27,551.79	27,557.79	1	<.01

Bold values indicate the model specification with lowest AIC estimate for each subscale.

**Figure 2:**
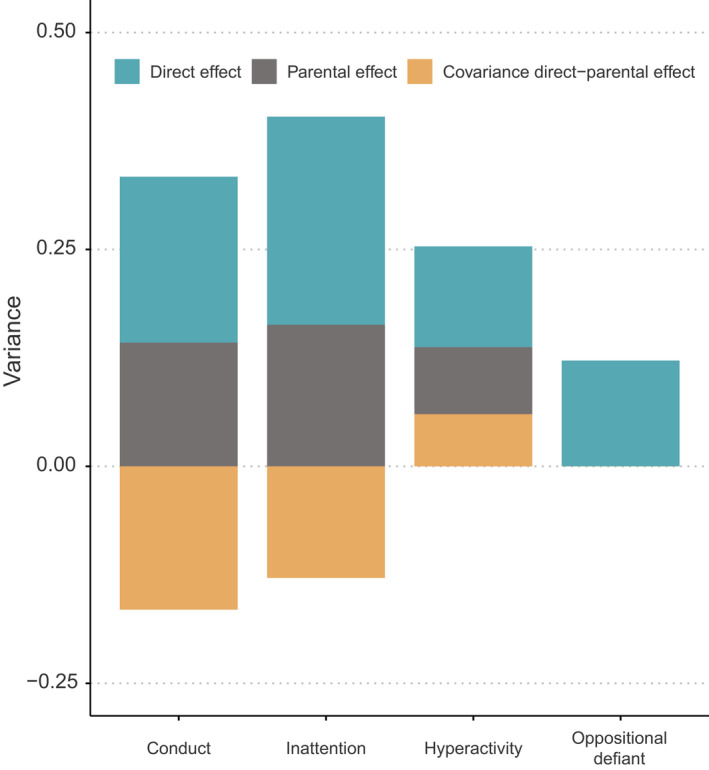
Variance decomposition for each subscale under the best fitting model [Colour figure can be viewed at wileyonlinelibrary.com]

Figure 2 shows the variance decomposition for the models with lowest AIC values for each subscale (a corresponding figure from the most general model is provided in Figure S1). Looking at the parameter estimates from these models, direct genetic effects accounted for 19.1% (*SE* = 6.3%) of the variance for conduct, 24% (*SE* = 8.2%) for inattention, 11.6% (*SE* = 7.9%) for hyperactivity and 12.2% (*SE* = 3.9%) for oppositional defiant.

In comparison, the combined indirect genetic effects from both parents accounted for 14.3% (*SE* = 6.1%) of the variance for conduct, 16.3% (*SE* = 7.9%) for inattention and 7.7% (*SE* = 7.9%) for hyperactivity. The model without any indirect genetic effects was favored for oppositional defiant.

The covariance between direct and indirect genetic effects was negative for conduct and inattention, reducing the variance by 16.5% (*SE* = 5.4%) and 12.9% (*SE* = 10%), respectively. This corresponds to a negative gene–environment correlation of −1.00 between direct and indirect genetic effects for conduct problems and a gene–environment correlation of −0.65 for inattention. A negative correlation implies that direct and indirect genetic effects to some extent counteract each other, decreasing the observed variation. For hyperactivity the covariance was positive, increasing the variance by 6% (*SE* = 9.8%), corresponding to a gene–environment correlation of 0.64. A positive correlation implies that direct and indirect genetic effects to some extent accompany each other, increasing the observed variation.

## Discussion

In summary, we find statistical support for a role of parental effects on externalizing disorders, in terms of both measures of model fit and substantial effect sizes. We could, however, not make any meaningful distinction between maternal and paternal genetic effects in the current dataset. Across subscales, direct genetic effects accounted for 12% to 24% of the variance, whereas indirect parental genetic effects accounted for 0% to 16%. Additionally, the covariance between direct and indirect parental effects, ranging from −0.17 to 0.06, indicates that neither heritability nor parenting can be properly understood without a simultaneous consideration of the other.

Across subscales, direct genetic effects accounted for 11% to 24% of the total variance. This is consistent with a heritable component underlying individual differences in externalizing problems, but lower than what is typically found in family studies (Bornovalova et al., 2010; Burt, 2009). The discrepancy between heritability estimates from SNP data and from family data has been discussed by others (Yang et al., 2017; Young, 2019; Young et al., 2018). Notably, heritability estimates depended on the inclusion of indirect parental genetic effects in the model. Due to the negative correlation with indirect genetic effects, heritability estimates are larger for conduct and inattention than when such effects are not modeled. The opposite pattern is seen for hyperactivity. In a comparison of twin and SNP heritability estimates, Cheesman et al. (2017) found larger discrepancies for childhood behaviour problems compared to height and cognition. Considering our results, the larger twin‐SNP heritability gap for behavior problems could be due to negative covariance with indirect parental genetic effects influencing behavior problems.

Overall, our results are consistent with the notion that parents contribute substantially to individual differences in childhood externalizing behaviors. In our analyses, interactive processes between parents and offspring through development are not specified but their aggregated effects are decomposed into components attributable to genetic variation in parents and in offspring and their covariance. The direct effect measures the combined effect of all genotyped SNPs in the offspring, regardless of whether these are mediated through multiple transactions with parental behaviors. Similarly, the parental genetic effects may ultimately represent a series of transactions with offspring behaviors. Thus, we cannot characterize the underlying processes, but we can make some broader observations.

First, indirect maternal and paternal genetic effects have a genetic basis in parents, but they necessarily contribute to variability in offspring through the environment. Therefore, we provide empirical support to theoretical models emphasizing the importance of the family environment for the development of externalizing behaviors (Hinshaw & Beauchaine, 2015).

Second, our results suggest a more important role of parents in the development of externalizing disorders than has been indicated in previous empirical investigations of specific risk factors. Hinshaw and Beauchaine (2015) use the principles of equifinality, and multifinality for describing the development of externalizing disorders. Equifinality implies that different causal pathways may lead to similar phenotypes, whereas multifinality implies that similar risk factors may lead to divergent phenotypes. Under this framework, simple ‘main effects’ models of specific risk factors are not expected to account for much of the observed differences in externalizing disorders. Our results demonstrate that genetic differences among parents are related to phenotypic differences in their offspring, irrespective of the underlying mechanisms. The results of this study may therefore help reconcile empirical findings of small effects of specific parental risk factors with theoretical models of externalizing disorders emphasizing the importance of parents.

Third, Burt (2009) reported that 23% of the variability in externalizing problems could be accounted for by environmental effects shared among siblings. Such effects may include parental behaviors, but also other aspects of the environment such as peers or schools. Plomin (2011) argued that experiences in the family may not be shared among siblings, but rather specific to the individual (Plomin & Daniels, 1987). Importantly, our approach provides a direct test of this view as we do not have to assume that all parental influences are shared among siblings. Our results only rely on demonstrating that there is an effect of parental genes on the trait of the children after accounting for direct effects of the children's own genes. However, our estimates are confined to parental effects that can be accounted for by the additive effects of genotyped SNPs, which is likely lower than the total additive effect (Yang et al., 2017). Moreover, the total indirect effect of parents may include genetic effects due to rare variants, non‐additive genetic effects and presumably an important environmental component. Therefore, our approach is limited to a conservative estimate of the total effect of parents.

In all results involving indirect genetic effects, we found that these effects were correlated with the direct genetic effect, rendering a gene–environment correlation (Plomin et al., 1977; Scarr & McCartney, 1983). This implies that the family environment is not independent of children's own genetic liability towards externalizing disorders. This has important implications for the study of family environments because it suggests that any naïve association with specific aspects of the family may be confounded by genetic effects and does not have a causal social interpretation. We observed that the correlation between direct and indirect genetic effects was negative for conduct and inattention, but positive for hyperactivity. We avoid trying to interpret these differences between point estimates because of the relatively large uncertainty of estimates and due to the correlations among the subscales. However, it is of interest to note that a strong negative correlation between direct and indirect maternal genetic effects have often been reported in the animal literature and has been the subject of much debate (Lee, 2002). A negative correlation implies that direct genetic effects and indirect genetic effects of parents tend to contribute to the trait in the opposite direction. Parents with children having an above average genetic propensity for these externalizing behaviors tend to also ‘reduce’ the behaviors towards the average, and vice versa. From an evolutionary perspective, such negative correlations may play an important role in the maintenance of genetic variation across generations (Räsänen & Kruuk, 2007). When a heritable trait is selected on in evolution, both genetic effects contributing to the behavior directly, and genetic effects contributing to the behavior indirectly through parental behaviors can evolve together. If counteracting parental effects tend to decrease variability due to direct genetic effects in offspring externalizing behaviors, this would lead to more children close to the average and less children with impairment due to extreme values on these behaviors. We are unaware of corresponding estimates related to psychological traits in humans but obtaining such estimates across different domains of behavior could lead to a better understanding of the transactional dynamics between parents and children.

There are several limitations that should be considered when interpreting the results of our study. As can be seen in Table 1, the likelihood values from alternative model specifications do not differ much. Consequently, the statistical support in favor of selecting any of the specific genetic models cannot be considered strong. Our main contribution is to demonstrate an indirect effect of parental genes, above the direct effect of children's own genes, and to show that these sources of variability are not independent. Precise quantification of the importance of either will require larger sample sizes.

Attrition from the MoBa study may plausibly be related to higher levels of externalizing symptoms, and thus correlated with genetic differences between families as well as children's levels of externalizing symptoms. Structured attrition of this type may introduce systematic differences between the study sample and the target population of the MoBa study (Biele et al., 2019). Unfortunately, it is difficult to evaluate the extent to which the current results depend on attrition and, thus, the generalizability of the current results remains unclear.

Measurements of children's externalizing symptoms were obtained by maternal reports. As, parents observe their children in a wide variety of contexts, maternal reports may provide more valid measurements than, for example measurements obtained from teachers or clinical interviews. However, such reports may be biased from characteristics of mothers, which could also bias estimates of indirect genetic effects. As we do not have measurements from independent raters, we cannot empirically investigate whether our results depend on such effects. Such validation studies would provide important additions to this study. Additionally, in the current analyses, we have treated the different subscales of externalizing problems separately, although they are in most accounts viewed as sharing underlying etiology (Nikolas, 2015). This makes it difficult to make comparison between different subscales. Further insights could likely be gained by considering structural models that explicitly accounts for the dependence structure. For example, according to Beauchaine and McNulty (2013), direct genetic effects are assumed to underlie a common liability for externalizing problems, whereas environmental influences are of greater importance for specific behaviors. Such assumptions could readily be incorporated in factor models, although it would likely be computationally challenging.

Finally, although our results support the existence of parental effects on child externalizing symptoms, the estimates may also capture population stratification and assortative mating. Future work should aim to estimate parental effects while accounting for assortative mating, which may be pronounced for neurodevelopmental disorders (Nordsletten et al., 2016). Indirect genetic effects from siblings are also a potential contribution to individual differences that may be correlated with parental effects. As can be seen in Table 1, most of the families in the study had several children in the household. Acknowledging these limitations, our results are consistent with the notion that parents contribute substantially to individual differences in childhood externalizing behaviors.

## Supporting information


**Table S1.** Distribution of missing responses within each subscale.
**Figure S1.** Variance decomposition for each subscale under the full ‘Differential parental’ model.
