# Supplementary material for: On the importance of parenting in externalizing disorders: an evaluation of indirect genetic effects in families
Source: J Child Psychol Psychiatry. 2022 Jul 2;63(10):1186–95. doi: 10.1111/jcpp.13654 (PMC9796091; doi:10.1111/jcpp.13654)

**Supporting information**

**MoBagenetics pre-imputation quality control and imputation**

Quality control exclusion criteria for individuals were: genotyping call rate <95%, or autosomal heterozygosity >4 standard deviations from the sample mean. Quality control exclusion criteria for SNPs (single nucleotide polymorphisms) were: ambiguous (A / T and C / G), genotyping call rate <98%, minor allele frequency <1%, or Hardy-Weinberg equilibrium P-value <1 × 10-6. Population stratification was assessed, using the HapMap phase 3 release 3 as a reference, by principal component analysis using EIGENSTRAT version 6.1.4. Visual inspection identified a homogenous population and individuals of non-European ancestries were removed based on principal component analysis of markers overlapping with available HapMap markers. The parent and offspring datasets were then merged into one dataset per genotyping batch, keeping only the SNPs that passed quality control in both datasets. Phasing was conducted using Shapeit2 release 837 and the duoHMM approach was used to account for the pedigree structure. Imputation was conducted using the Haplotype reference consortium (HRC) release 1-1 as the genetic reference panel. The Sanger Imputation Server was used to perform the imputation with the Positional Burrows-Wheeler Transform (PBWT). The phasing and imputation were conducted separately for each genotyping batch. More detailed information about the cohorts, quality control and imputation can be found at https://github.com/folkehelseinstituttet/mobagen https://github.com/folkehelseinstituttet/mobagen.

**Response data**

Table S1 displays the number of missing responses within each subscale. A complete set of responses were obtained for most participants. Of those not responding to all items, it was most common to miss one item.

Table S1. Distribution of missing responses within each subscale.

|  |  | Missing responses | | | | | | | | | |
| --- | --- | --- | --- | --- | --- | --- | --- | --- | --- | --- | --- |
| Scale |  | 0 | 1 | 2 | 3 | 4 | 5 | 6 | 7 | 8 | 9 |
| Conduct |  | 11397 | 95 | 15 | 28 | 2* | 17* | 0* | 0* | 6* | NA |
| Inattention |  | 11315 | 189 | 20 | 1 | 2 | 3* | 1* | 2* | 3* | 24* |
| Hyperactivity |  | 11358 | 142 | 17 | 8 | 2 | 2* | 2* | 0* | 2* | 27* |
| Oppositional defiant |  | 11358 | 151 | 10 | 2 | 2* | 1* | 1* | 6* | 29* | NA |

*Refers to those that were excluded because of too many missing responses.

**Variance decomposition**

Figure S1 shows the variance decomposition for each subscale under the full “Differential parental” model. When considering the combined maternal and paternal effects, the pattern of results looks relatively similar to the results from the models with the lowest AIC values, presented in the main text. There are some differences between the size of the maternal and paternal indirect genetic effects, and the corresponding covariances with direct genetic effects. Additionally, these results imply both a maternal and paternal genetic effect with negative covariances with direct genetic effects for Oppositional defiant, which was not implied from the model with lowest AIC values. However, these estimates do not have any strong statistical support as indicated from the model comparison presented in the main text. These results should therefore be interpreted with care.

*Figure S1. Variance decomposition for each subscale under the full “Differential parental” model.*


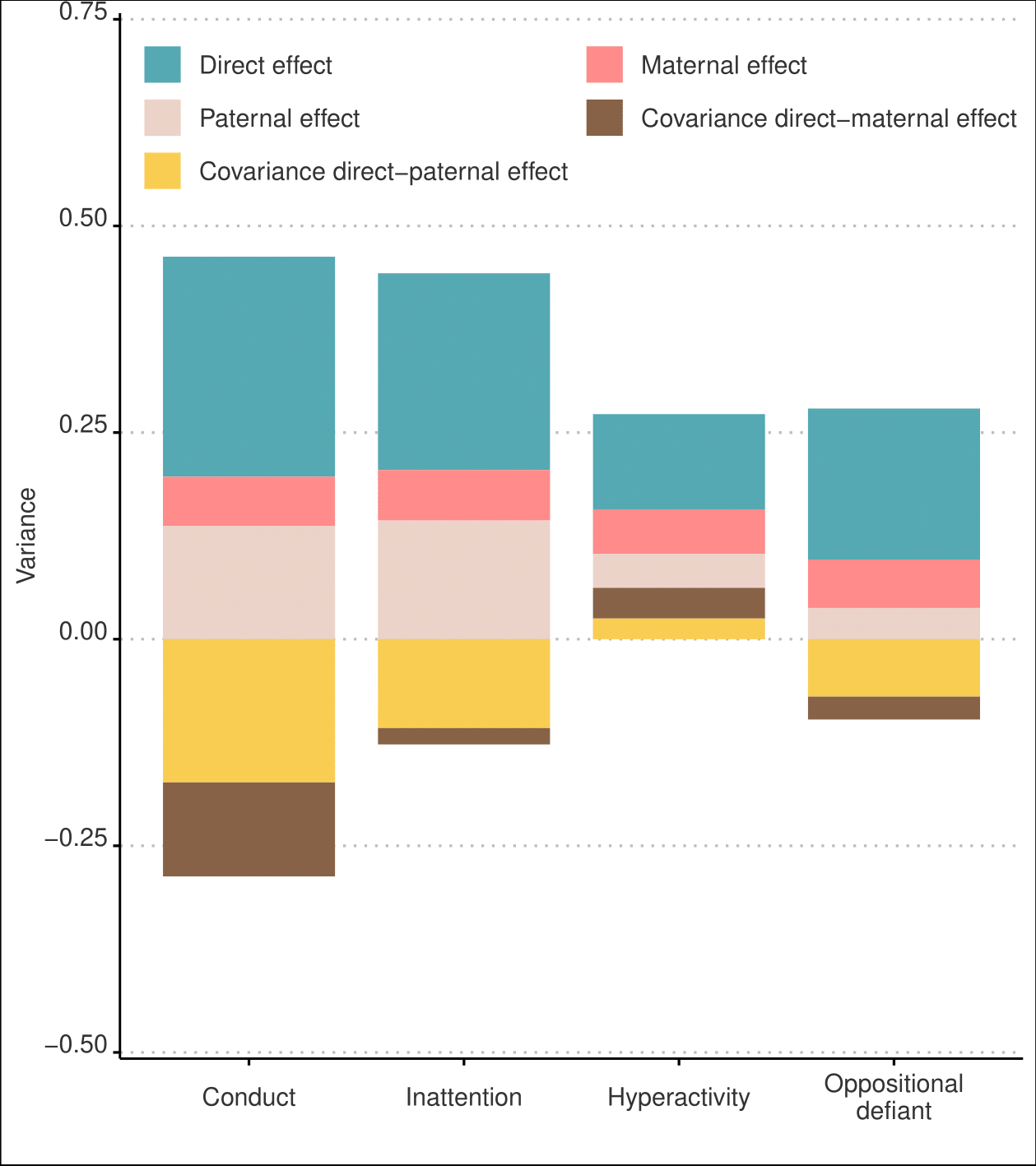

Supplement: Supplementary file 1 — Table S1. Distribution of missing responses within each subscale. Figure S1. Variance decomposition for each subscale under the full ‘Differential parental’ model. [file JCPP-63-1186-s001.docx]
